# Cervical brachalgia: Assessment by cervical CT epidurography post transforaminal injection

**DOI:** 10.2349/biij.5.2.e9

**Published:** 2009-04-01

**Authors:** P Emberton, SP Tan

**Affiliations:** 1 Queens Medical Centre, Nottingham University Hospitals, Nottingham, United Kingdom; 2 National University Malaysia Medical Centre (UKMMC), Cheras, Kuala Lumpur, Malaysia

**Keywords:** Cervical brachalgia, transforaminal injections (TFI), adjunct cervical CT epidurography (CCTE)

## Abstract

Adjunct cervical CT epidurography (CCTE) can be used to image impingement in patients with cervical brachalgia undergoing fluoroscopic-guided cervical transforaminal injection (TFI) of steroid/local anaesthetic where magnetic resonance imaging (MRI) is contraindicated. CCTE images of the 9 patients on whom the authors performed CCTE post TFI over 6 years from 1998 to 2003 were retrospectively reviewed. CCTE is able to provide good images of the cervical spinal canal and its contents. CCTE may be an alternative imaging method for impingement in patients with cervical brachalgia contraindicated for MRI.

## INTRODUCTION>

Surgery is a treatment option for cervical brachalgia. Some patients do not experience symptomatic relief post surgery. Follow-up imaging of these patients with MRI is often difficult in the presence of artefact-producing metallic implants.

Some of these patients may undergo diagnostic or therapeutic tranforaminal injection (TFI) of steroid/local anaesthetic to confirm the level of impingement. In this group of patients an adjunct cervical CT epidurography (CCTE) was performed immediately following the TFI. During the TFI, low osmolar iodinated contrast media (LOCM) was injected as positive contrast and air was injected as negative contrast. The authors looked at the spinal canal and its contents for evidence of impingement as a possible cause for cervical brachalgia. CT epidurography without TFI of LOCM as contrast has been used to understand deformation of the lumbar dural sac post epidural injections [1]. CCTE post TFI, to the authors' knowledge has not been previously described in the English medical literature.

Therefore, the authors propose CCTE as an alternative imaging method for impingement in patients with cervical brachalgia who are absolutely contraindicated for MRI.

## MATERIALS AND METHODS

This is a retrospective review of the case notes of all 9 patients done over 6 years from 1998 to 2003 who underwent CCTE post TFI. They were contraindicated for MRI because they either had artefact producing cervical metallic implants or were severely claustrophobic.

The technique of fluoroscopic-guided cervical TFI was described by Bush [2]. Under fluoroscopic screening using aseptic technique, a 25G 3 ½-inch spinal needle was passed into the cervical exit foramen, ensuring the needle tip was posterior and lateral to the vertebral artery position. Subsequent injection of 0.5-1 ml of LOCM (Niopam 200, Iopamidol, Bracco s.p.a. Milan, Italy) as positive contrast was used to confirm the epidural location of the needle tip. This location was evidenced by a satisfactory outline of the root sheath and epidural spread, and also excluded intravascular spread. Usually, a mixture of 1.5 ml lignocaine hydrochloride 1% (Lignocaine, Pfizer, Perth, Australia) and triamcinolone acetonide 40mg/ml (Kenalog 40; Bristol-Myers-Squibb, New Jersey, USA) was subsequently injected for pain relief and to reduce local inflammation. An extension of the standard technique was that approximately 3 ml of air was injected as negative contrast prior to removing the needle.

CCTE was then performed with a four-slice CT scanner (GE Lightspeed, Milwaukee, Wisconsin, USA). The scanning was done with 0.75 mm collimation and 1.25 mm table movement and reconstructed at 1 mm thick sections with an increment of 0.5 mm. Images in both bone and soft tissue algorithms were reviewed.

## RESULT

**Figure 1:**
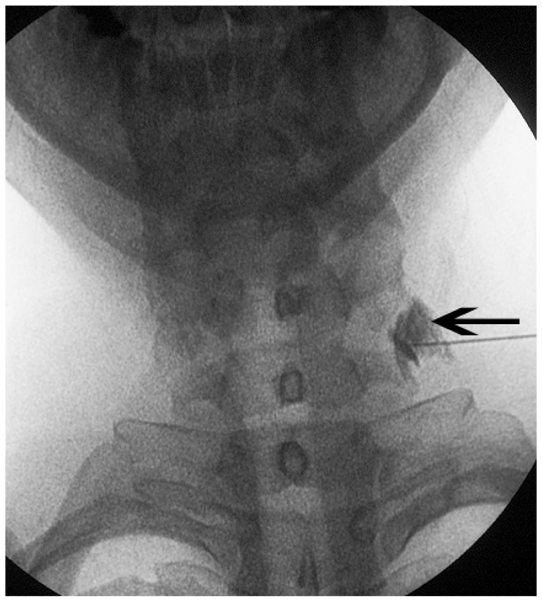
Fluoroscopic anteroposterior (AP) view of a TFI. Positive contrast in the epidural location (black arrow) confirms proper positioning of the needle tip.

**Figure 2:**
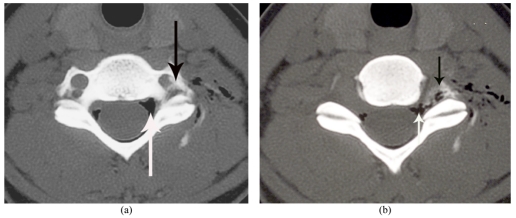
(a) Axial images of CCTE post a left C6/C7 TFI just above the level of the C6/C7 exit foramen in a 28-year-old female patient with left C7 radiculopathy. Positive contrast (LOCM) and negative contrast (air) in the epidural space are indicated by black and white arrows, respectively. The spinal canal and exit foramina bilaterally were normal in this patient. (b) Axial images of CCTE at the level of the C6/C7 exit foramen of the same patient. Positive contrast (LOCM) and negative contrast (air) in the epidural space are indicated by black and white arrows, respectively. The spinal canal and exit foramina bilaterally were normal in this patient.

There were no complications from the TFI. All 9 patients tolerated the adjunct CCTE well. A sample patient’s findings are illustrated. This was a 28-year-old lady with left C7 radiculopathy. Figure 1 shows a fluoroscopic AP view of the cervical spine with positive contrast outlining the epidural space around the needle tip. The patient's CCTE images (fig 2) suggest a normal spinal canal and exit foramina at these levels. The patient did not undergo surgery and unfortunately no follow-up of this patient could be obtained.

## DISCUSSION

Findings have been limited by the small number of patients involved in this study. No positive pathological findings on CCTE have been documented in all these patients. Unfortunately, the authors were unable to trace the images or follow up any of their other patients due to the long time lapse from when the cases were done to the time of this write-up.

CCTE may prove to be an alternative imaging technique for post surgical patients with cervical brachalgia in whom MRI may be unhelpful due to the presence of artefacts from cervical metallic implants. As illustrated, CCTE is able to provide good images of the cervical spine and its contents.

The risk of air embolism in TFI is important and should be emphasized. It is avoided by the administration of positive contrast to confirm satisfactory epidural location of the needle tip. In a study with 5,334 patients who underwent epidurography and therapeutic epidural injections, four complications occurred which included a hypotensive episode, a small dorsal epidural haematoma without cord or neural compression, vasovagal response and an episode of tachycardia 12 hours after an uneventful epidural injection [3]. None of the complications required surgical intervention and all were self-limited with regard to symptoms and imaging manifestations. The authors advocated that the proceduralist take direct responsibility for follow-up care during 5 to 7 days after injection.

CCTE post TFI is, at present, only performed in patients where MRI is contraindicated. This explains the small number of patients recruited over the 6 years despite having a large spinal unit at the centre. This technique needs further assessment to determine its clinical value.
